# Small GTPase Rab17 Regulates the Surface Expression of Kainate Receptors but Not α-Amino-3-hydroxy-5-methyl-4-isoxazolepropionic Acid (AMPA) Receptors in Hippocampal Neurons via Dendritic Trafficking of Syntaxin-4 Protein*

**DOI:** 10.1074/jbc.M114.550632

**Published:** 2014-06-03

**Authors:** Yasunori Mori, Mitsunori Fukuda, Jeremy M. Henley

**Affiliations:** From the ‡ School of Biochemistry, University of Bristol, Bristol BS8 1TD, United Kingdom and; the § Laboratory of Membrane Trafficking Mechanisms, Department of Developmental Biology and Neurosciences, Graduate School of Life Sciences, Tohoku University, Aobayama, Aoba-ku, Sendai, Miyagi 980-8578, Japan

**Keywords:** Ionotropic Glutamate Receptor, Membrane Trafficking, Neuron, Rab Proteins, SNARE Proteins

## Abstract

Glutamate receptors are fundamental for control synaptic transmission, synaptic plasticity, and neuronal excitability. However, many of the molecular mechanisms underlying their trafficking remain elusive. We previously demonstrated that the small GTPase Rab17 regulates dendritic trafficking in hippocampal neurons. Here, we investigated the role(s) of Rab17 in AMPA receptor (AMPAR) and kainate receptor (KAR) trafficking. Although Rab17 knockdown did not affect surface expression of the AMPAR subunit GluA1 under basal or chemically induced long term potentiation conditions, it significantly reduced surface expression of the KAR subunit GluK2. Rab17 co-localizes with Syntaxin-4 in the soma, dendritic shaft, the tips of developing hippocampal neurons, and in spines. Rab17 knockdown caused Syntaxin-4 redistribution away from dendrites and into axons in developing hippocampal neurons. Syntaxin-4 knockdown reduced GluK2 but had no effect on GluA1 surface expression. Moreover, overexpression of constitutively active Rab17 promoted dendritic surface expression of GluK2 by enhancing Syntaxin-4 translocation to dendrites. These data suggest that Rab17 mediates the dendritic trafficking of Syntaxin-4 to selectively regulate dendritic surface insertion of GluK2-containing KARs in rat hippocampal neurons.

## Teaser

**Background:** Rab17 regulates dendritic trafficking in hippocampal neurons, but the cargo molecules are unknown.

**Results:** Using GluK2 and GluA1 as markers, we show that Rab17 or Syntaxin-4 knockdown reduces dendritic surface expression of KARs but not AMPARs.

**Conclusion:** Rab17 mediates membrane insertion of GluK2 but not GluA1 via dendritic trafficking of Syntaxin-4.

**Significance:** Syntaxin-4 specifically regulates GluK2 trafficking.

## Introduction

Glutamate receptors are critical for excitatory synaptic transmission and plasticity. They are broadly classified into ionotropic and metabotropic types, and ionotropic glutamate receptors are further categorized into NMDA, AMPA, and kainate receptors (1). AMPARs^3^ are tetrameric complexes of combinations of four separate subunits (GluA1–4). They are highly mobile proteins that undergo constitutive- and activity-dependent translocation to, and removal from, synapses (2). Crucially, changes in the number and properties of functional synaptic AMPARs results in the long term potentiation (LTP) or long term depression of synaptic efficacy (3).

KARs are also tetrameric assemblies of combinations of five possible subunits (GluK1–5). Presynaptic KARs modulate neurotransmitter release; postsynaptic KARs mediate excitatory neurotransmission, and extrasynaptic KARs are involved in controlling neuronal excitability (4, 5). Importantly, KARs participate in the regulation of neuronal network activity and are involved in processes ranging from neuronal development to neurodegeneration and neuronal cell death (4, 5).

Multiple proteins have been identified as participating in AMPAR trafficking. These include AP-4 and KIF5, which are involved in transporting AMPARs from the soma to dendrites (6, 7); SNARE complex family proteins Syntaxin-3, Syntaxin-4, and SNAP-47 are implicated in AMPAR surface insertion (8, 9); and small GTPase Rab and Arf family proteins (*i.e.* Rab11 and Arf1) play roles in AMPAR internalization and/or recycling (10, 11). In contrast, relatively few interacting proteins have been implicated in KAR trafficking, although Rab11 is involved in KAR recycling and SNAP-25 in internalization (12, 13). Furthermore, the similarities and differences between the mechanisms and pathways that regulate AMPAR and KAR trafficking and surface expression have not been well defined.

Rab proteins are small GTPases that are conserved in all eukaryotes. They mediate trafficking steps, including vesicle budding, translocation, docking to specific membranes, and fusion (14, 15). Rab17 was originally described as an epithelial cell-specific protein that regulates polarized trafficking (16, 17). However, we showed previously that Rab17 is also expressed in mouse brain where it is involved in the regulation of dendritic morphogenesis and postsynaptic development of hippocampal neurons (18). Interestingly, Rab17 is the only reported somatodendritic-compartmentalized Rab, but its functional roles in dendritic trafficking remain unclear.

We investigated the possible relationship between Rab17 and AMPAR and KAR trafficking in hippocampal neurons. Rab17 knockdown did not affect trafficking of the AMPAR subunit GluA1, but it markedly reduced surface expression of the KAR subunit GluK2. Surprisingly, GluK2 and Rab17 do not co-localize. Rather, Rab17 is highly co-localized with Syntaxin-4 in somatodendritic compartments of developing neurons and in spines of mature neurons. Rab17 knockdown decreased Syntaxin-4 in dendrites and increased mis-targeted Syntaxin-4 in axons of developing neurons. Syntaxin-4 knockdown reduced GluK2 surface expression but did not affect GluA1. Furthermore, Syntaxin-4 knockdown prevented the increase in surface GluK2 induced by expression of a constitutively active form of Rab17. Taken together, these results suggest Rab17 mediates polarized trafficking of Syntaxin-4 and that dendritic Syntaxin-4 is important for surface insertion of GluK2-containing KARs but not GluA1-containing AMPARs in the somatodendritic compartment of rat hippocampal neurons.

## EXPERIMENTAL PROCEDURES

### 

#### 

##### Antibodies

Commercially obtained antibodies were as follows: anti-c-Myc (9E10) mouse monoclonal antibody (Santa Cruz Biotechnology); anti-calcium/calmodulin-dependent protein kinase II α (6G9) mouse monoclonal antibody, anti-GAPDH (6C5) mouse monoclonal antibody, and anti-MAP2 chick polyclonal antibody (Millipore Corp.); anti-GFP (clones 7.1 and 13.1) mouse monoclonal antibody (Roche Applied Science); anti-Syntaxin-4 rabbit polyclonal antibody (Synaptic Systems); IRDye®680RD/800CW-conjugated anti-mouse/rabbit IgG donkey antibody (LI-COR Biotechnology); horseradish peroxidase (HRP)-conjugated anti-mouse antibody (Sigma); and Cy2/Cy3/Cy5 or Alexa Fluor® 488/594/647-conjugated anti-mouse/rabbit/chick/sheep IgG donkey antibody (Jackson ImmunoResearch). Anti-Rab17 rabbit polyclonal and anti-Myc sheep antibodies were prepared as described previously (18, 19).

##### Plasmid Construction

cDNAs encoding the mouse Syntaxin-1a, Syntaxin-4, or rat Syntaxin-4 were amplified from adult mouse brain, testis, or adult rat brain cDNA by PCR. Purified PCR products were directly inserted into the pGEM-T Easy vector (Promega) or pCR-Blunt TOPO II vector (Invitrogen). The cDNAs were then subcloned into the BamHI/EcoRI site of the pCMV-Myc vector (20) to create pCMV-Myc-Stx1, pCMV-Myc-Stx4, and pCMV-Myc-ratStx4. Rab17-shRNA (18), rat Syntaxin4-shRNA-1 (21-base target site, 5′-caagtgtaactcaatgcagtc-3′), which was slightly modified as described in Jurado *et al.* (9), and rat Syntaxin4-shRNA-2 (19-base target site, 5′-agacaattcggcgactat-3′), which was described in Kennedy *et al.* (8), were subcloned into BamHI/HindIII site of pSilencer-CMV-EGFP vector (21) or BbsI/EcoRI site of pFIV-H1/U6-puro vector (System Bioscience) to create pSilencer-CMV-EGFP-shRab17, pFIV-shRab17, pFIV-shStx4-1, and pFIV-shStx4-2.

A mutant of Syntaxin-4 resistant to shRNA-1 (Syntaxin-4^SR^) was produced from mouse Syntaxin-4 cDNA as described previously (18) using the following oligonucleotides (substituted nucleotides are shown in uppercase): Syntaxin-4-SR-5′ primer, 5′-gcaatttgtcgagctcatcaaTaaAtgCaaTAGTatgcaAAGcgaataccgagagaagaatg-3′ and Syntaxin-4-SR-3′ primer, 5′-cattcttctctcggtattcgCTTtgcatACTAttGcaTttAttgatgagctcgacaaattgc-3′. The DNA of Syntaxin-4^SR^ or Rab17^SR^ (18) were subcloned into the BamHI/EcoRI site or BglII/EcoRI site of the pCMV-Myc vector to create pCMV-Myc-Stx4^SR^, pCMV-Stx4^SR^, and pCMV-Rab17^SR^. pEGFP-Rab17, pmCherry-Rab17, pEGFP-Rab17-Q77L, pCAG-Control, pCAG-Myc-Rab17-Q77L, pCMV-Myc-Stx2, and pCMV-Myc-Stx3 were prepared as described previously (18, 22). pCDNA3-Myc-GluA1, pCDNA3-Myc-GluK2, and pCDNA3-SEP-Myc-GluK2 were also prepared as described previously (11, 23). pEGFP-C2 was from Clontech.

##### Hippocampal Neuron Culture, Cell Line Culture, and Transfections

Rat embryonic hippocampal neuronal cultures were prepared from E18 Wistar rats. Neurons were then plated at a density of 75,000–100,000 onto 22-mm glass coverslips in a 6-well dish coated with 1 mg/ml poly-l-lysine (Sigma). The culture medium was composed of Neurobasal medium supplemented with 10% horse serum, 2% B27, 1% GlutaMAX, and 50 units/ml penicillin/streptomycin (all reagents from Invitrogen). On the 2nd day, the media were changed for Neurobasal medium supplemented with 2% B27 and 0.6% GlutaMAX, and 2.5 μm cytosine-β-d-arabinofuranoside (Sigma) was add to the medium after the 4th day of plating. Plasmid DNAs were transfected into the neurons by using Lipofectamine®2000 (Invitrogen) according to the manufacturer's instructions.

HEK293T cells and Neuro2A cells were cultured in Dulbecco's modified Eagle's medium (DMEM, Lonza) supplemented with 10% fetal bovine serum (FBS), 50 units/ml penicillin/streptomycin. The cells were plated onto a 6-well plate. Plasmid DNAs were transfected into JET-PEI (Poly-Plus transfection) according to the manufacturer's instructions.

##### Immunostaining

Neurons were fixed for 10 min with 4% paraformaldehyde (PFA), which was diluted by phosphate-buffered saline (PBS) from 16% PFA (Electron Microscopy Sciences) at room temperature. After permeabilizing the cells with 0.1% Triton X-100 or 0.01% digitonin (for endogenous Syntaxin-4 staining) in PBS for 10 min, they were blocked with the blocking buffer (10% horse serum in PBS) for 1 h. The neurons were then immunostained for primary antibody for 1 h at room temperature or overnight at 4 °C after which they were incubated for 1 h with fluorescence-conjugated secondary IgG at room temperature. The neurons were examined for fluorescence with an LSM510 META confocal laser-scanning microscope (Zeiss), and the images were processed with Adobe Photoshop CS software. Fluorescent intensity or the number of fluorescent dots were quantified with ImageJ software (version 1.42q; National Institutes of Health), and Pearson's correlation coefficient was determined manually by using the co-localization indices plug-in (24) to the ImageJ software program. Representative images of neurons are shown in each figure.

##### Immunoblotting

Cell lysates were subjected to SDS-PAGE and proteins transferred to PVDF membrane (Millipore) by electroblotting. The blots were blocked with 0.3% skim milk and 0.2% Tween 20 in PBS, and after incubating them with a primary antibody, they were washed with PBS containing 0.2% Tween 20 and incubated with a peroxidase-conjugated secondary antibody. Immunoreactive bands were detected by enhanced chemiluminescence or fluorescence with exposure to x-ray film or ODYSSEY® FC dual-imaging system (LI-COR Biotechnology) for quantifying the bands by using LI-COR Image Studio software.

##### Chem-LTP

Chem-LTP was induced as essentially described previously with slight modification (25). Briefly, at 18 DIV, neuronal culture was changed into extracellular solution (ECS: 150 mm NaCl, 2 mm CaCl_2_, 5 mm KCl, 10 mm HEPES (pH 7.4), 30 mm glucose) containing 0.5 μm tetrodotoxin. After 5 min in ECS, neurons were treated with 200 μm glycine (chem-LTP condition) or without glycine (basal condition) for 3 min in ECS and then incubated ECS containing tetrodotoxin without glycine for 5 min.

##### GluA1 and GluK2 Surface Expression

GluA1 and GluK2 were surface immunostained using Myc-GluA1, Myc-GluK2, or SEP-GluK2 as described previously with slight modifications (23, 25). Neurons were fixed in 4% PFA in PBS for 10 min under the nonpermeabilized conditions. For visualizing surface Myc-GluA1 or Myc-GluK2, the neurons were incubated with Myc antibody in the blocking buffer (1:500 dilution) for 20 min and then Cy3-conjugated secondary antibody (1:1000 dilution) for 20 min. Surface SEP-GluK2 was visualized by incubating neurons with Alexa Fluor® 555-conjugated anti-GFP antibody (Invitrogen) in the blocking buffer (1:2000 dilution) for 5 min and then Cy3-conjugated secondary antibody (1:1000 dilution) for 1 h. The neurons were additionally fixed with 4% PFA in PBS for 10 min and permeabilized, and they were subjected to immunostaining with anti-Myc antibody (1:200 dilution), anti-GFP antibody (1:200 dilution), and Cy5- or Cy2-conjugated secondary antibody again to visualize total levels. Surface expression levels were quantified by dividing the surface fluorescence intensity by the total fluorescence intensity.

##### Quantification of Somatic, Axonal, and Dendritic Distributions

Protein compartmentalization was quantified by setting image thresholds to exclude pixels outside the neuron. After subtracting the background intensity values from each image before quantification, the integrated fluorescence intensity of the protein in the entire neuron or just the region of soma, axon, or dendrite region was obtained. The proportion (%) of the protein in the region was calculated by dividing the fluorescence intensity of the region by the fluorescence intensity of the entire neuron.

##### Statistical Analyses

Student's unpaired *t* test was used to evaluate every result for statistical significance in comparison with the results obtained in control. The single asterisk and double asterisk in the bar charts indicate a *t* test *p* value of <0.05 and <0.0025, respectively, and comparisons that yielded a *p* value >0.05 are indicated by NS (not significant).

## RESULTS

### 

#### 

##### Rab17 Is Required for Surface Insertion of GluK2 but Not GluA1

**FIGURE 1.**
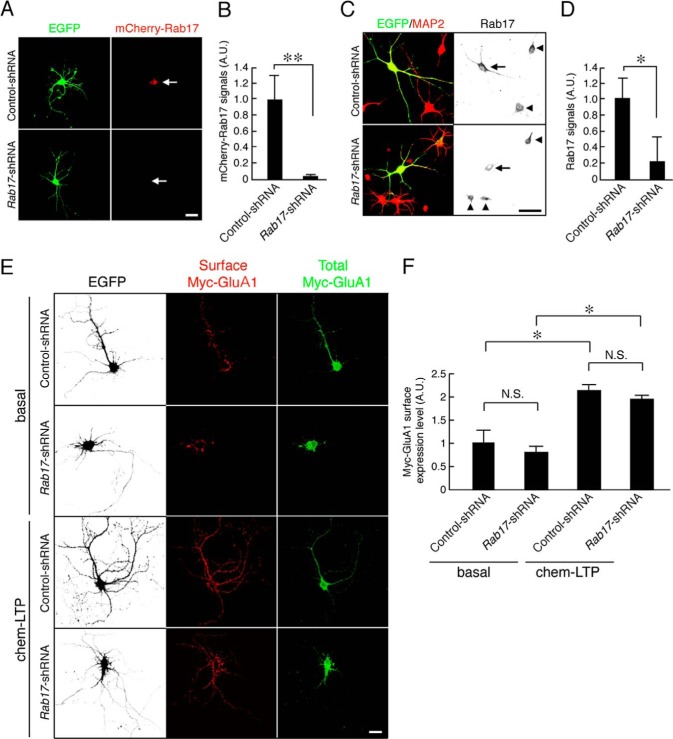
**Rab17 is not required for the surface expression of GluA1 in rat hippocampal neurons.** *A*, representative images of mCherry-Rab17-expressing neurons in the presence and absence of *Rab17*-shRNA. At 6 DIV, rat hippocampal neurons were transfected with pmCherry-Rab17 together with pSilencer-CMV-EGFP-Control or pSilencer-CMV-EGFP-shRab17, and at 8 DIV, the neurons were fixed. The *arrows* indicate the as-transfected neurons. *Bar,* 10 μm. *B,* quantification of the mCherry-Rab17 of control-shRNA-transfected neurons (*n* = 10) and *Rab17*-shRNA-transfected neurons (*n* = 10) shown in *A. A.U.*, arbitrary units. **, *p* < 0.0025. *C,* representative images of endogenous Rab17 in the presence and absence of *Rab17*-shRNA. At 8 DIV, rat hippocampal neurons were transfected with pSilencer-CMV-EGFP-Control or pSilencer-CMV-EGFP-shRab17, and at 11 DIV, the neurons were fixed and subjected to immunocytochemistry with antibodies against Rab17 (*black*) and MAP2 (*red*). The *arrows* and *arrowheads* indicate the as-transfected neurons and untransfected neurons, respectively. *Bar,* 10 μm. *D,* quantification of the Rab17 of control-shRNA-transfected neurons (*n* = 10) and *Rab17*-shRNA-transfected neurons (*n* = 10) shown in *C. A.U.*, arbitrary units. *, *p* < 0.025. *E,* representative images of surface expression level of Myc-GluA in the *Rab17*-shRNA-transfected neurons under basal or chem-LTP conditions. At 10 DIV, rat hippocampal neurons were transfected with pCDNA3-Myc-GluA together with pSilencer-CMV-EGFP-Control or pSilencer-CMV-EGFP-shRab17, and at 18 DIV, the neurons were stimulated or not with 200 μm glycine for 3 min (basal or chem-LTP conditions). After stimulation, the neurons were fixed and subjected to immunocytochemistry with antibodies against Myc (surface, *red* and total, *green*). GFP image replaced black in this figure. *Bar,* 10 μm. *F,* quantification of the surface expression level of Myc-GluA1 of control-shRNA-transfected neurons under basal conditions (*n* = 10); *Rab17*-shRNA-transfected neurons under basal conditions (*n* = 10); control-shRNA-transfected neurons under chem-LTP conditions (*n* = 10); and *Rab17*-shRNA-transfected neurons under chem-LTP conditions (*n* = 10) shown in *E*. The rate of surface expression level of Myc-GluA1 was calculated by dividing the surface Myc-GluA1 fluorescence intensity by the total Myc-GluA1 fluorescence intensity. *A.U.,* arbitrary units. *, *p* < 0.025. *N.S.*, not significant.

We first investigated the effects of knocking down Rab17 on surface expression of the AMPAR subunit GluA1 under basal and glycine-stimulated chem-LTP conditions. *Rab17*-shRNA prevented expression of exogenously expressed mCherry-Rab17 and knocked down endogenous Rab17 by almost 80% in rat hippocampal neurons (levels compared with control-shRNA were 3.0 ± 2.3% for mCherry-Rab17 and 21.6 ± 31.1% for endogenous Rab17) (Fig. 1, *A–D*).

Rab17 knockdown did not significantly affect surface levels of Myc-GluA1 under basal (*Rab17*-shRNA, 79.8 ± 14.1% of the control) or chem-LTP conditions (control-shRNA, 216.0 ± 10.6%; *Rab17*-shRNA, 194.3 ± 9.5% compared with basal levels in control neurons) in 18 DIV hippocampal neurons (Fig. 1, *E* and *F*). These results indicate that Rab17 is not involved in GluA1 trafficking in rat hippocampal neurons.

**FIGURE 2.**
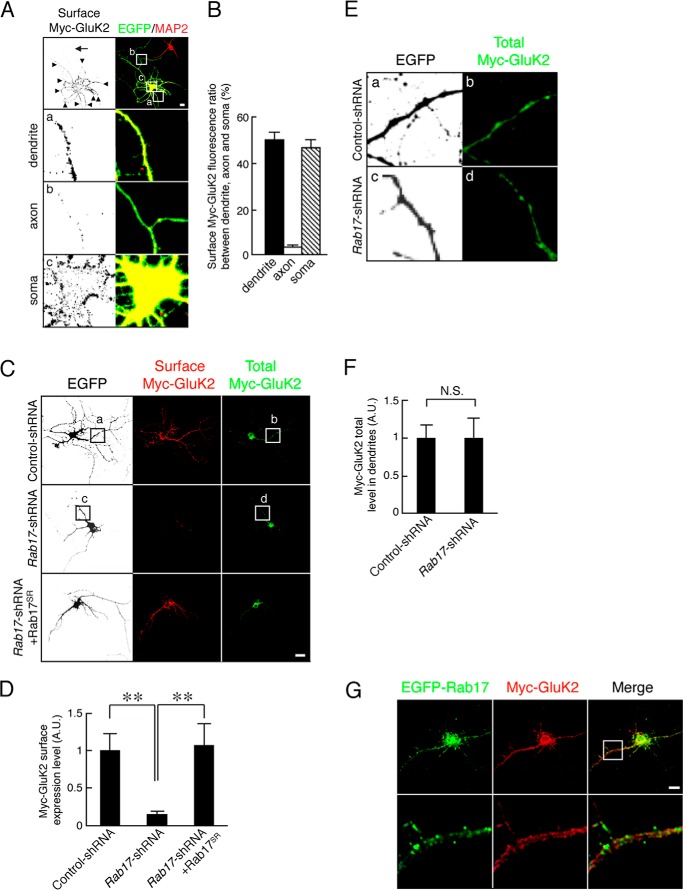
**Rab17 is required for the surface expression of GluK2 without affecting its traffic to the dendrites.** *A,* representative images of the distribution of surface-expressed Myc-GluK2 in developing hippocampal neurons. At 8 DIV, rat hippocampal neurons were co-transfected with pCDNA3-Myc-GluK2 and pEGFP-C2. At 14 DIV, the neurons were fixed and subjected to immunocytochemistry with antibodies against Myc (surface; *black*) and MAP2 (*red*) as a dendrite marker. The *arrows* and *arrowheads* point to axons and dendrites, respectively. *Panels a–c* are magnified views of the *boxed areas* in the *top right panels. Bar,* 10 μm. *B,* quantification of the ratio of surface-expressed Myc-GluK2 between soma, dendrite, and axon (*n* = 10) as shown in *A. C*, representative images of surface expression level of Myc-GluK2 in the *Rab17*-shRNA-transfected neurons. At 8 DIV, rat hippocampal neurons were transfected with pCDNA3-Myc-GluK2 together with pSilencer-CMV-EGFP-Control, pSilencer-CMV-EGFP-shRab17, or pSilencer-CMV-EGFP-shRab17 and pCMV-Rab17^SR^. At 14 DIV, neurons were fixed and subjected to immunocytochemistry with antibodies against Myc (surface, *red* and total, *green*). GFP image was replaced black in this figure. *Bar,* 10 μm. *D,* quantification of the surface expression level of Myc-GluK2 of control-shRNA-transfected neurons (*n* = 10) and *Rab17*-shRNA-transfected neurons (*n* = 10) as shown in *C*. The rate of surface expression level of Myc-GluK2 was calculated by dividing the surface Myc-GluK2 fluorescence intensity by the total Myc-GluK2 fluorescence intensity. *A.U.*, arbitrary units. **, *p* < 0.0025. *E,* magnified views of the boxed areas in *panels a–d* of *C. Bar,* 10 μm. *F,* quantification of the total Myc-GluK2 fluorescence intensity in the dendrites of control-shRNA-transfected neurons (*n* = 10) and *Rab17*-shRNA-transfected neurons (*n* = 10) as shown in *E*. Total level of Myc-GluK2 in the dendrites was calculated by dividing the Myc-GluK2 fluorescence intensity by the dendrite length. *A.U.,* arbitrary units. *N.S.*, not significant. *G,* representative images of Myc-GluK2 and EGFP-Rab17 in neurons. At 8 DIV, rat hippocampal neurons were co-transfected with pEGFP-Rab17 and pCDNA3-Myc-GluK2. At 14 DIV, the neurons were fixed and subjected to immunocytochemistry with antibodies against Myc (*red*). The *bottom panels* are magnified views of the boxed areas in the *upper panels. Bar,* 5 μm.

We next examined whether Rab17 is involved KAR surface expression. Because it has been reported that the KAR subunit GluK2 is present at both the pre- and postsynapse (4), we assessed the ratio of surface distribution of Myc-GluK2 in the soma, dendrites, and axons of 14 DIV hippocampal neurons. Myc-GluK2 was predominantly surface-expressed in the soma and dendrite with much less present in axons (ratio of surface-expressed Myc-GluK2 50.7 ± 1.5% in dendrite, 3.2 ± 0.9% in axon, and 46.6 ± 3.6% in soma; Fig. 2, *A* and *B*). Thus, almost all of the surface signal of Myc-GluK2 can be attributed to the somatodendritic compartment under these conditions. Knockdown of Rab17 dramatically reduced GluK2 surface expression in the rat hippocampal neurons at 14 DIV (to 14.7 ± 4.1% of the control neurons; Fig. 2, *C* and *D*). This loss was completely rescued by re-expression of shRNA-resistant mutant Rab17^SR^ (to 106.7 ± 28.8% of the control neurons) (Fig. 2, *C* and *D*).

Interestingly, Rab17 knockdown did not change the total fluorescence intensity of GluK2 in the dendrites (to 103.0 ± 24.7% of the control neurons; Fig. 2, *E* and *F*). Furthermore, Rab17 did not co-localize with GluK2 in dendrites (Fig. 2*G*). Thus, Rab17 is involved in GluK2 surface expression but is not required for the transport of GluK2 from the soma to the dendrite.

##### Rab17 Co-localizes with Syntaxin-4 in Hippocampal Neurons

**FIGURE 3.**
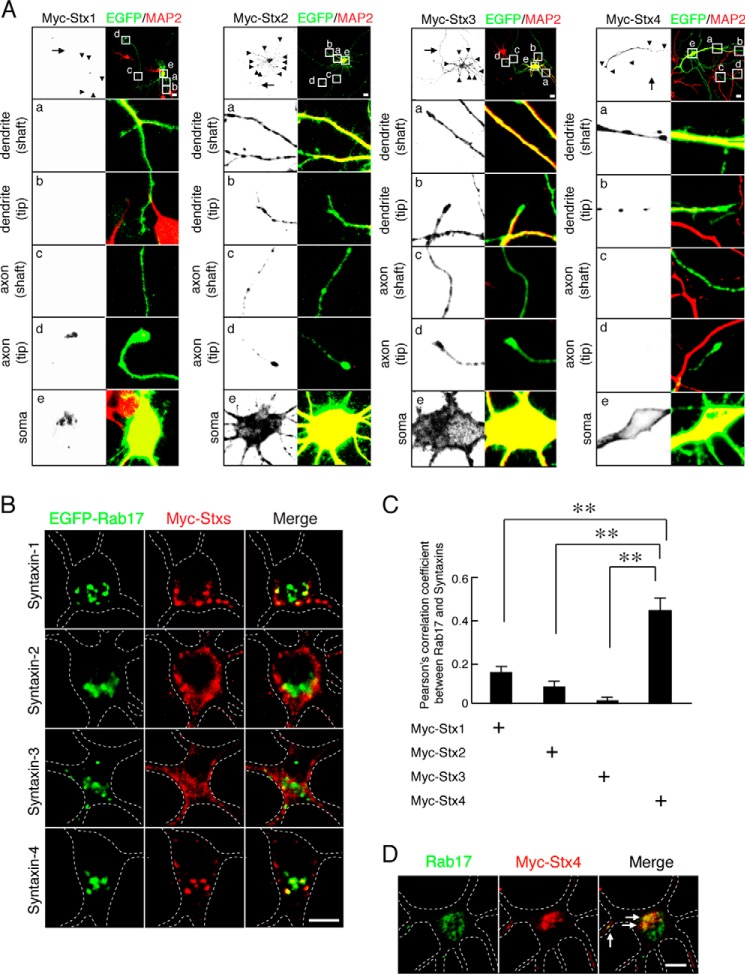
**Rab17 co-localizes with Syntaxin-4 in the soma.** *A*, representative images of plasma membrane-associated Syntaxins in developing neurons. At 8 DIV, mouse hippocampal neurons were transfected with pCMV-Myc-Stx1 (*left panel*), pCMV-Myc-Stx2 (*2nd panel* from the *left*), pCMV-Myc-Stx3 (*3rd panel* from the *left*), or pCMV-Myc-Stx4 (*right panel*), and at 11 DIV, the neurons were fixed and subjected to immunocytochemistry with antibodies against Myc (*black*) and MAP2 (*red*). The *arrows* and *arrowheads* point to axons and dendrites, respectively. The lower four *panels a–d* are magnified views of the *boxed areas* in the *top right panels. Bar,* 10 μm. *B,* representative images of Rab17 and plasma membrane-associated Syntaxins at the soma in developing neurons. At 8 DIV, mouse hippocampal neurons were transfected with pEGFP-Rab17 together with pCMV-Myc-Stx1 (*top panel*), pCMV-Myc-Stx2 (*2nd panel*), pCMV-Myc-Stx3 (*3rd panel*), or pCMV-Myc-Stx4 (*bottom panel*), and at 11 DIV, the neurons were fixed and subjected to immunocytochemistry with antibodies against Myc (*red*) and MAP2. The *dashed lines* indicate dendritic shafts identified as MAP2-positive areas. *Bar,* 5 μm. *C,* quantification of the Pearson's correlation coefficient between EGFP-Rab17 and Myc-Syntaxin-1 (*n* = 10), Myc-Syntaxin-2 (*n* = 10), Myc-Syntaxin-3 (*n* = 10), and Myc-Syntaxin-4 (*n* = 10), as shown in *A.* **, *p* < 0.0025. *D,* representative images of endogenous Rab17 and Myc-Syntaxin-4 in the soma at developing neurons. At 8 DIV, hippocampal neurons were transfected with pCMV-Myc-Stx4, and at 11 DIV, the neurons were fixed and subjected to immunocytochemistry with antibodies against Rab17 (*green*), Myc (*red*), and MAP2. The *dashed lines* indicate MAP2-positive areas, and the *arrows* indicate the co-localization points. *Bar,* 5 μm.

These data suggest that Rab17 regulates GluK2 surface expression indirectly via some intermediate protein(s). It has been reported that Syntaxin-4, but not Syntaxin-1, Syntaxin-2, or Syntaxin-3, localizes at the postsynaptic plasma membrane in hippocampal neurons (8). We therefore assessed co-localization of Rab17 with each of these Syntaxin isoforms. First, we checked the localization of Myc-tagged Syntaxin-1, -2, -3, and -4 in 11 DIV hippocampal neurons. Myc-Syntaxin-1 was present in the soma and at axon tips but was rarely detected in the axonal shaft or in the dendritic shaft or tip (Fig. 3*A, left panels*). Myc-Syntaxin-2 and Myc-Syntaxin-3 were present in all compartments (Fig. 3*A, middle panels*). Myc-Syntaxin-4 mainly localized at the soma, dendritic shaft, and tip, with no detectable staining in axons (Fig. 3*A, right panels*). Rab17 confined to the somatodendritic compartment (18) and EGFP-Rab17 is highly co-localized with Myc-Syntaxin-4 but not with Syntaxin-1, Syntaxin-2, or Syntaxin-3 in the soma in 11 DIV hippocampal neurons (Fig. 3*B*). Pearson's correlation coefficients between Rab17 and the syntaxin paralogues were as follows: Syntaxin-1, 0.15 ± 0.03; Syntaxin-2, 0.08 ± 0.03; Syntaxin-3, 0.01 ± 0.02; and Syntaxin-4, 0.45 ± 0.06 (Fig. 3*C*).Importantly, there was also extensive co-localization between endogenous Rab17 also co-localized with Myc-Syntaxin-4 at the soma (Fig. 3*D*).

**FIGURE 4.**
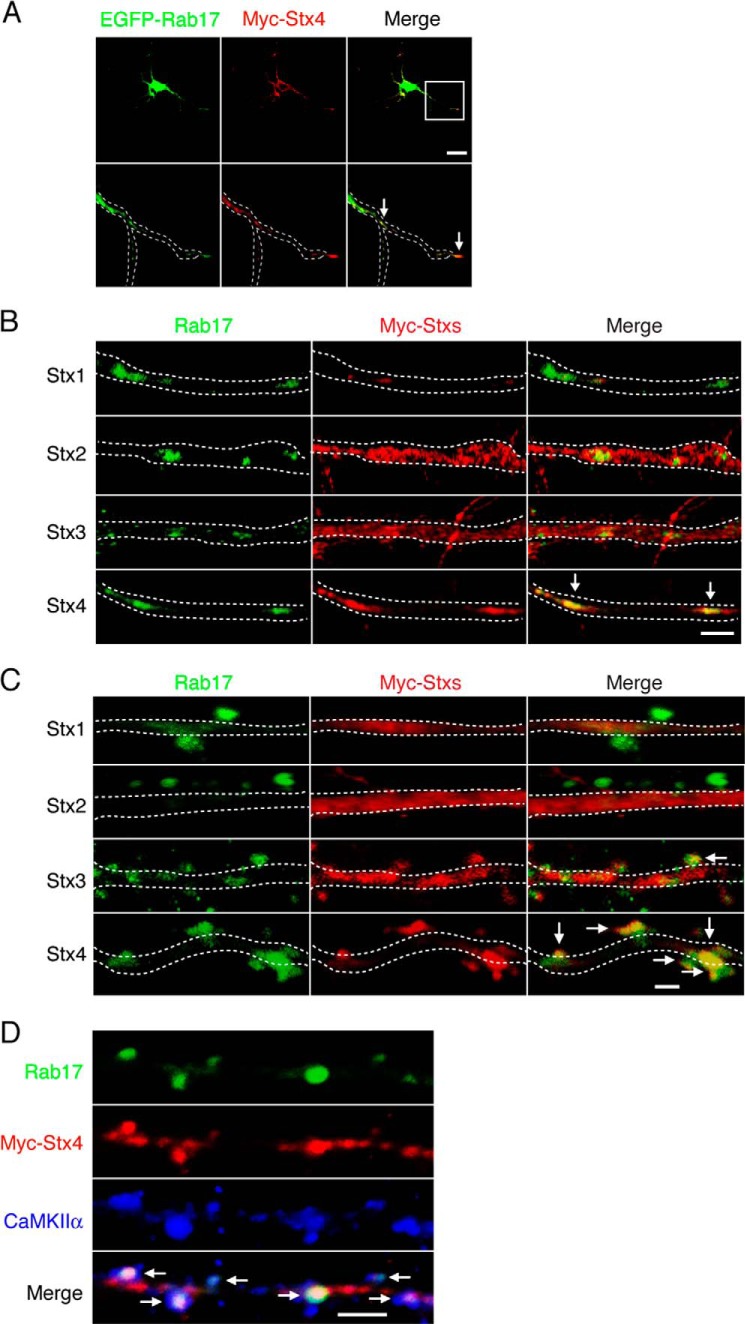
**Rab17 co-localizes with Syntaxin-4 in the dendritic shaft, tip, and spine.** *A,* representative images of EGFP-Rab17 and Myc-Syntaxin-4 in the dendrite at developing neurons. At 8 DIV, mouse hippocampal neurons were co-transfected with pEGFP-Rab17 and pCMV-Myc-Stx4, and at 11 DIV, the neurons were fixed and subjected to immunocytochemistry with antibodies against Myc (*red*) and MAP2. The *bottom panels* are magnified views of the *boxed area* in the *top right panel*, and the *dashed lines* indicate dendritic shafts identified as MAP2-positive areas. The *arrows* indicate the co-localization points. *Bar,* 10 μm. *B,* representative images of Rab17 and plasma membrane-associated Syntaxins in the distal dendrite at developing neurons. At 8 DIV, mouse hippocampal neurons were transfected with pCMV-Myc-Stx1 (*top panels*), pCMV-Myc-Stx2 (*2nd panel*), pCMV-Myc-Stx3 (*3rd panel*), or pCMV-Myc-Stx4 (*bottom panel*), and at 11 DIV, the neurons were fixed and subjected to immunocytochemistry with antibodies against Rab17 (*green*), Myc (*red*), and MAP2. The *dashed lines* indicate dendritic shafts identified as MAP2-positive areas. The *arrows* indicate the co-localization points. *Bar,* 5 μm. *C,* representative images of Rab17 and plasma membrane-associated Syntaxins in the spine at matured neurons. At 8 DIV, mouse hippocampal neurons were transfected with pCMV-Myc-Stx1 (*top panels*), pCMV-Myc-Stx2 (*2nd panel*), pCMV-Myc-Stx3 (*3rd panel*), or pCMV-Myc-Stx4 (*bottom panel*), and at 21 DIV, the neurons were fixed and subjected to immunocytochemistry with antibodies against Rab17 (*green*), Myc (*red*), and MAP2. The *dashed lines* indicate dendritic shafts identified as MAP2-positive areas. The *arrows* indicate the co-localization points. *Bar,* 5 μm. *D,* representative images of Rab17 and Myc-Syntaxin-4 in the mature neurons. At 8 DIV, rat hippocampal neurons were transfected with pCMV-Myc-Stx4, and at 24 DIV, the neurons were fixed and subjected to immunocytochemistry with antibodies against Rab17 (*green*), Myc (*red*), and calcium/calmodulin-dependent protein kinase II α (*CaMKII*α) (a spine marker; *blue*). *Bar,* 5 μm. The *arrows* indicate the co-localization points.

Rab17 and Myc-Syntaxin-4, but not Myc-Syntaxin-1, -2, or -3, puncta also co-localize in the dendrites (Fig. 4, *A* and *B*). Because Rab17 accumulates at dendritic spines in mature hippocampal neurons (18), we next investigated possible co-localization with Myc-Syntaxin-1, -2, -3, and -4 at spines in 21 DIV mature neurons. As shown in Fig. 4*C*, Myc-Syntaxin-1 and Myc-Syntaxin-2 were only present in the dendritic shaft (Fig. 4*C, top rows*). Myc-Syntaxin-3 was present in the dendritic shaft, and there was some overlap with Rab17-positive protuberances (Fig. 4*C, 3rd row*). In contrast, Myc-Syntaxin-4 displayed strong co-localization at the Rab17-positive protuberances with relatively little present in the dendritic shaft (Fig. 4*C, bottom row*). Furthermore, the Rab17 and Myc-Syntaxin-4 signals co-localize with the postsynaptic spine marker calcium/calmodulin-dependent protein kinase II α (Fig. 4*D*), indicating that they accumulate at spines.

##### Rab17 Is Required for the Polarized Distribution of Syntaxin-4 in Developing Hippocampal Neurons

**FIGURE 5.**
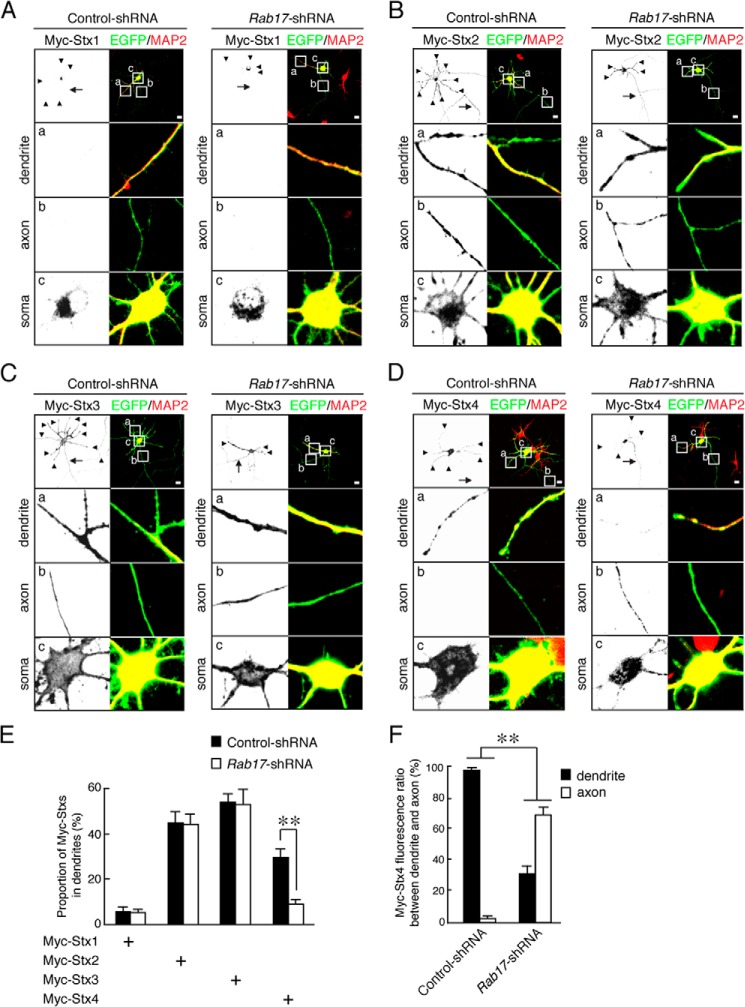
**Rab17 is required for polarized trafficking of Syntaxin-4 to the dendrite.** *A–D,* representative images of plasma membrane-associated Syntaxins in the *Rab17*-shRNA-transfected neurons. At 8 DIV, mouse hippocampal neurons were transfected with pCMV-Myc-Stx1 (*A*), pCMV-Myc-Stx2 (*B*), pCMV-Myc-Stx3 (*C*), or pCMV-Myc-Stx4 (*D*) together with pSilencer-CMV-EGFP-Control or pSilencer-CMV-EGFP-shRab17, and at 11 DIV, the neurons were fixed and subjected to immunocytochemistry with antibodies against Myc (*black*), GFP (*green*), and MAP2 (*red*). The *arrows* and *arrowheads* point to axons and dendrites, respectively. The *lower three panels a–c* are magnified views of the *boxed areas* in the *top right panels. Bar,* 10 μm. *E,* quantification of the proportion of Myc-Syntaxin-1 (*n* = 10), Myc-Syntaxin-2 (*n* = 10), Myc-Syntaxin-3 (*n* = 10), and Myc-Syntaxin-4 (*n* = 10) in the control neurons and *Rab17*-shRNA-transfected neurons at the dendrites, as shown in *A–D*. The rate of translocated level of Myc-Syntaxin-4 was calculated by dividing the dendrite Myc-Syntaxin-4 fluorescence intensity by the total Myc-Syntaxin-4 fluorescence intensity. **, *p* < 0.0025. *F,* quantification of the fluorescence ratio of Myc-Syntaxin-4 between dendrite and axon in the control neurons and *Rab17*-shRNA transfected neurons, as shown in *D*. **, *p* < 0.0025.

We next investigated whether Rab17 is involved in Syntaxin-4 trafficking. As expected, Rab17 knockdown did not alter the localizations of Myc-Syntaxin-1, Syntaxin-2, or Syntaxin-3 in 11 DIV hippocampal neurons (dendritic Myc-Syntaxin-1, 5.7 ± 1.9 and 5.2 ± 1.5% in control and Rab17-knockdown neurons, respectively; dendritic Myc-Syntaxin-2, 44.8 ± 4.8 and 44.0 ± 4.5% in control and Rab17-knockdown neurons, respectively; dendritic Myc-Syntaxin-3, 53.8 ± 3.9 and 52.9 ± 6.3% in control neurons and Rab17-knockdown neurons, respectively; Fig. 5, *A–E*). In stark contrast, however, Rab17 knockdown markedly reduced dendritic Myc-Syntaxin-4 (Myc-Syntaxin-4 in the dendrites, 29.7 ± 3.5% of the control neurons *versus* 9.0 ± 2.0% of the Rab17-knockdown neurons) (Fig. 5, *D* and *E*). Additionally, Rab17 knockdown significantly reduced the dendritic and increased axonal localization of Myc-Syntaxin-4 (Myc-Syntaxin-4 ratio between dendrites and axons = 97.6 ± 1.2 and 2.4 ± 1.2% in control neurons and 31.2 ± 4.6 and 68.8 ± 4.9% in the Rab17-knockdown neurons; Fig. 5, *D* and *F*).

**FIGURE 6.**
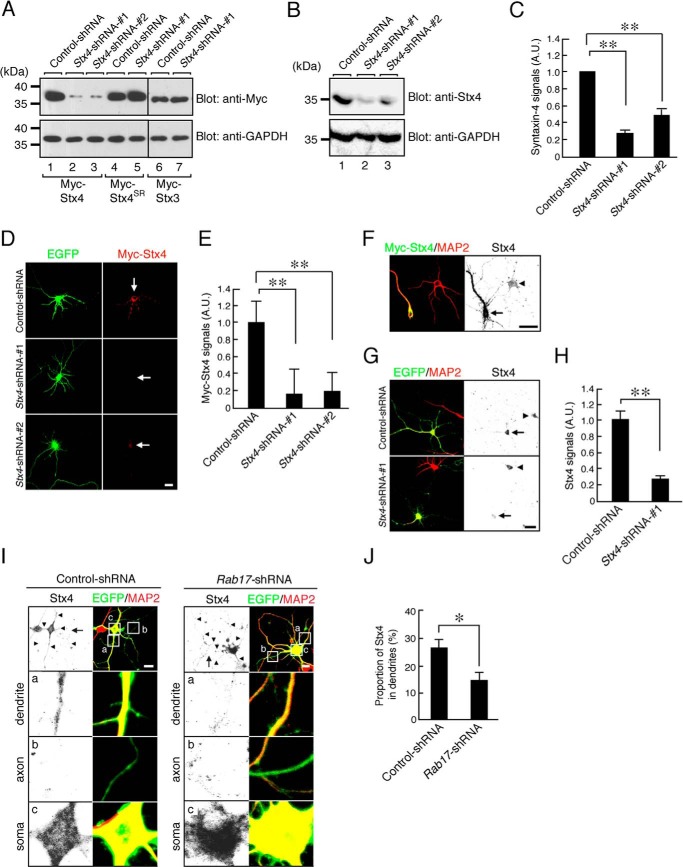
**Rab17 is required for dendritic trafficking of endogenous Syntaxin-4.** *A,* HEK293T cells were transfected with pFIV-Control (*lanes 1, 4,* and *6*), pFIV-shStx4-1 (*lanes 2, 5,* and *7*), or pFIV-shStx4-2 (*lane 3*) together with pCMV-Myc-ratStx4 (*lanes 1–3*), pCMV-Myc-Stx4^SR^ (*lanes 4* and *5*), or pCMV-Myc-Stx-3 (*lanes 6* and *7*). Two days after transfection, the cells were lysed and subjected to immunoblot analysis with anti-Myc antibody (*upper panel*) and anti-GAPDH antibody (*lower panel*). *B,* Neuro2A cells were transfected with pFIV-Control (*lane 1*), pFIV-shStx4-1 (*lane 2*), or pFIV-shStx4-2 (*lane 3*). Two days after transfection the cells were lysed and subjected to immunoblot analysis with anti-Syntaxin-4 antibody (*upper panel*) and anti-GAPDH antibody (*lower panel*). *C,* quantification of endogenous Syntaxin-4 of control-shRNA, *Stx4*-shRNA-1 and *Stx4*-shRNA-2 as shown in *B. A.U.*, arbitrary units. **, *p* < 0.0025. *D,* representative images of Myc-Syntaxin-4-expressing neurons in the presence and absence of *Stx4*-shRNA. At 8 DIV, rat hippocampal neurons were transfected with pCMV-Myc-ratStx4 together with pFIV-Control, pFIV-shStx4-1, or pFIV-shStx4-2, and at 11 DIV, the neurons were fixed. The neurons were stained by Myc (*red*). *Bar,* 10 μm. *E,* quantification of the Myc-Syntaxin-4 of control-shRNA-transfected neurons (*n* = 10), *Stx4*-shRNA-1-transfected neurons (*n* = 10), and *Stx4*-shRNA-2-transfected neurons (*n* = 10) as shown in *B. A.U.*, arbitrary units. **, *p* < 0.0025. *F,* representative images of Myc-Syntaxin-4-expressing neurons stained by Syntaxin-4 antibody. At 8 DIV, rat hippocampal neurons were transfected with pCMV-Myc-Stx4, and at 11 DIV, the neurons were fixed. The neurons were stained by Myc (*green*), Syntaxin-4 (*black*), and MAP2 (*red*). *Bar,* 10 μm. *G,* representative images of endogenous Syntaxin-4 in the presence and absence of *Stx4*-shRNA. At 8 DIV, rat hippocampal neurons were transfected with pFIV-Control and pFIV-shStx4-1, and at 11 DIV, the neurons were fixed. The neurons were stained by Syntaxin-4 (*black*) and MAP2 (*red*). *Bar,* 10 μm. *H,* quantification of endogenous Syntaxin-4 of control-shRNA-transfected neurons (*n* = 10) and *Stx4*-shRNA-1-transfected neurons (*n* = 10) shown in *G. A.U.,* arbitrary units. **, *p* < 0.0025. *I,* representative images of endogenous Syntaxin-4 in the *Rab17*-shRNA-transfected neurons. At 8 DIV, mouse hippocampal neurons were transfected with pSilencer-CMV-EGFP-Control or pSilencer-CMV-EGFP-shRab17, and at 11 DIV, the neurons were fixed and subjected to immunocytochemistry with antibodies against Syntaxin-4 (*black*), GFP (*green*), and MAP2 (*red*). The *arrows* and *arrowheads* point to axons and dendrites, respectively. The *lower three panels a–c* are magnified views of the *boxed areas* in the *top right panels. Bar,* 10 μm. *J,* quantification of the proportion of endogenous Syntaxin-4 in the control neurons (*n* = 10) and *Rab17*-shRNA-transfected neurons (*n* = 10) at the dendrites, shown in *I*. The rate of translocated level of Syntaxin-4 was calculated by dividing the dendrite Syntaxin-4 fluorescence intensity by the total Syntaxin-4 fluorescence intensity. *, *p* < 0.025.

We used immunocytochemistry to define the effects of Rab17 knockdown on endogenous Syntaxin-4. To validate the specificity of the anti-Syntaxin-4 antibody in immunostaining, we constructed *Stx4*-shRNA-1 (9) and *Stx4*-shRNA-2 (8) to specifically knock down Syntaxin-4. Both shRNAs dramatically decreased both exogenously expressed Myc-Syntaxin-4 (Fig. 6*A*) and endogenous Syntaxin-4 levels in Neuro2A cells (*Stx4*-shRNA-1 reduced endogenous Syntaxin-4 levels to 27.6 ± 3.2% and *Stx4*-shRNA-2 reduced levels to 48.7 ± 6.3% compared with control-shRNA; Fig. 6, *B* and *C*). We also checked that both shRNAs were effective in hippocampal neurons (*Stx4*-shRNA-1 reduced levels to 16.0 ± 29.0% and *Stx4*-shRNA-2 reduced levels to 19.0 ± 22.4% compared with control; Fig. 6, *D* and *E*). Furthermore, enhanced anti-Syntaxin-4 antibody immunostaining was observed in hippocampal neurons expressing Myc-Syntaxin-4 (Fig. 6*F*), and decreased staining was observed in Syntaxin-4 knockdown neurons (*Stx4*-shRNA-1 reduced levels to 27.5 ± 6.3% compared with control-shRNA; Fig. 6, *G* and *H*). These results rigorously characterize the specificity and utility of the anti-Syntaxin-4 antibody and validate its use to monitor changes in endogenous Syntaxin-4 in neurons.

Interestingly, Rab17 knockdown resulted in the somatic accumulation of endogenous Syntaxin-4 with a consequent reduction in dendrites in 11 DIV hippocampal neurons (Syntaxin-4 in the dendrites, 26.0 ± 3.1% of the control neurons *versus* 14.1 ± 2.8% of the Rab17-knockdown neurons) (Fig. 6, *I* and *J*). However, unlike Myc-Syntaxin-4, endogenous Syntaxin-4 was not mis-targeted to axons in the Rab17 knockdown neurons (Fig. 6*I*). These results demonstrate that Rab17 is required for the translocation of Syntaxin-4 to the dendrites in hippocampal neurons.

##### Syntaxin-4 Is Necessary for GluK2 but not GluA1 Surface Insertion

**FIGURE 7.**
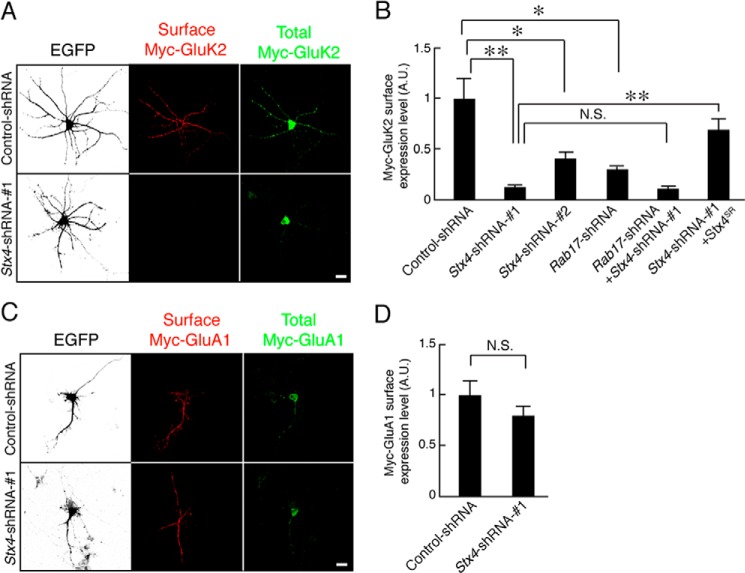
**Syntaxin-4 is necessary for the surface expression of GluK2.** *A,* representative images of surface expression level of Myc-GluK2 in the *Syntaxin-4*-shRNA-transfected neurons. At 8 DIV, rat hippocampal neurons were transfected with pCDNA3-Myc-GluK2 and pEGFP-C2 together with pFIV-Control or pFIV-shStx4-1, and at 11 DIV, the neurons were fixed and subjected to immunocytochemistry with antibodies against Myc (surface, *red* and total, *green*). GFP image was replaced black in this figure. *Bar,* 10 μm. *B,* quantification of the surface expression level of Myc-GluK2 of control-shRNA-transfected neurons as indicated (*n* = 10 for all conditions). At 8 DIV, rat hippocampal neurons were transfected with pCDNA3-Myc-GluK2 and pEGFP-C2 together with pFIV-Control, pFIV-shStx4-1, pFIV-shStx4-2, pFIV-shRab17, pFIV-shStx4-1, and pFIV-shRab17, or pFIV-shStx4-1 and pCMV-Stx4^SR^, and at 11 DIV, the neurons were fixed and subjected to immunocytochemistry with antibodies against Myc (surface, *red* and total, *green*). Surface expression level of Myc-GluK2 was calculated by dividing the surface Myc-GluK2 fluorescence intensity by the total Myc-GluA1 fluorescence intensity. *A.U.*, arbitrary units. **, *p* < 0.0025; *, *p* < 0.025. *N.S.*, not significant. *C,* representative images of surface expression level of Myc-GluA1 in the *Syntaxin-4*-shRNA-transfected neurons. At 8 DIV, rat hippocampal neurons were transfected with pCDNA3-Myc-GluA1 and pEGFP-C2 together with pFIV-Control or pFIV-shStx4-1, and at 11 DIV, the neurons were fixed and subjected to immunocytochemistry with antibodies against Myc (surface *red* and total, *green*). GFP image was replaced black in this figure. *D,* quantification of the surface expression level of Myc-GluA1 of control-shRNA-transfected neurons (*n* = 10) and *Stx4*-shRNA-1-transfected neurons (*n* = 10) as shown in *C*. Surface expression level of Myc-GluA1 was calculated by dividing the surface Myc-GluA1 fluorescence intensity by the total Myc-GluA1 fluorescence intensity. *A.U.*, arbitrary units. *N.S.*, not significant.

Syntaxin-4 knockdown also markedly reduced Myc-GluK2 surface expression in 11 DIV hippocampal neurons (11.6 ± 2.5 and 40.5 ± 6.2% of control surface Myc-GluK2 for *Stx4*-shRNA-1 and *Stx4*-shRNA-2, respectively; Fig. 7, *A* and *B*). Moreover, GluK2 surface expression in the combined Syntaxin-4- and Rab17-knockdown neurons is almost the same as knockdown of Syntaxin-4 alone (*Stx4*-shRNA-1 and *Rab17*-shRNA, 10.1 ± 2.3% of Myc-GluK2 of the control neurons). Surface expression of GluK2 in Syntaxin-4-knockdown neurons was rescued by re-expression of shRNA-resistant Syntaxin-4^SR^ (*Stx4*-shRNA-1 and Syntaxin-4^SR^, 69.2 ± 10.5% of Myc-GluK2 of the control neurons). As expected, knockdown of Syntaxin-4 did not significantly alter surface expression of the AMPAR subunit Myc-GluA1 at 11 DIV (*Stx4*-shRNA-1, 80.2 ± 10.0% of Myc-GluA1 of the control neurons; Fig. 7, *C* and *D*). These results are consistent with Rab17 and Syntaxin-4 working via the same pathway to regulate GluK2 surface expression but are not involved in GluA1 surface expression.

##### Constitutively Active Rab17 Promotes KAR Surface Expression by Enhancing Dendritic Localization of Syntaxin-4

**FIGURE 8.**
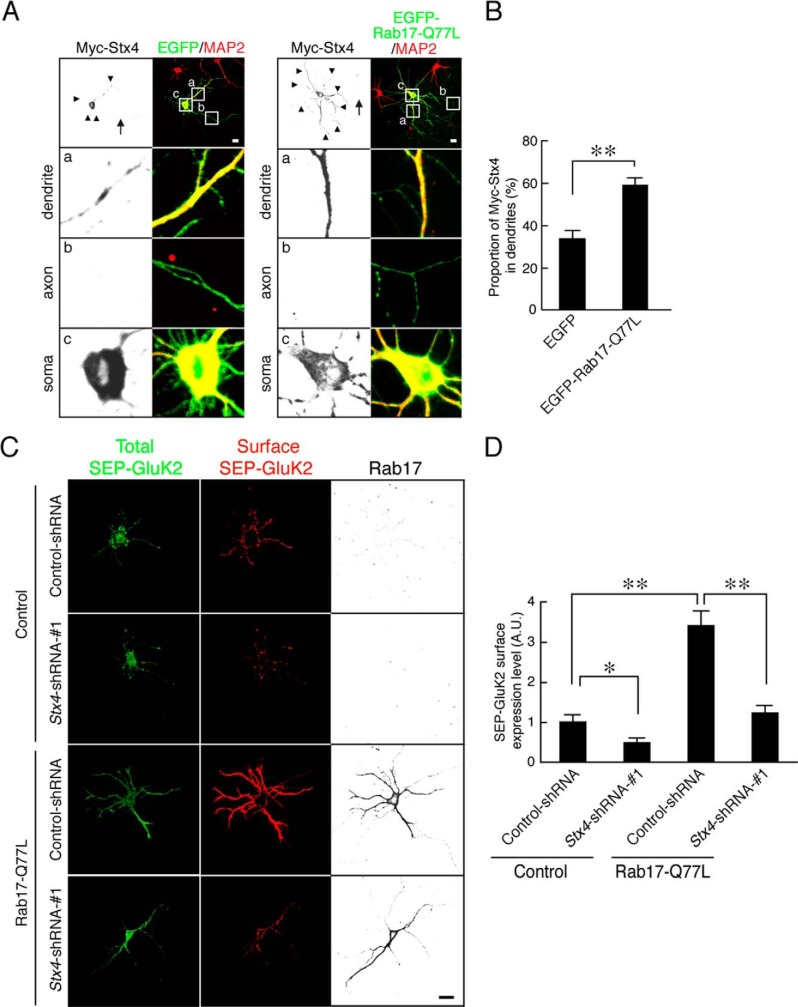
**Active form of Rab17 promotes surface expression of GluK2 by enhancing Syntaxin-4 translocation to dendrites.** *A,* representative images of Myc-Syntaxin-4 in active form Rab17-expressing neurons. At 8 DIV, rat hippocampal neurons were transfected with pCMV-Myc-Stx4 together with pEGFP-C2 or pEGFP-Rab17-Q77L. At 11 DIV, the neurons were fixed and subjected to immunocytochemistry with antibodies against Myc (*black*) and MAP2 (*green*). The *bottom three panels a–c* are magnified views of the *boxed areas* in the *top right panels. Bar,* 10 μm. *B,* quantification of the proportion of Myc-Syntaxin-4 in the dendrites in the presence of EGFP (*n* = 10) and EGFP-Rab17-Q77L as shown in *A*. The rate of translocated level of Myc-Syntaxin-4 was calculated by dividing the dendrite Myc-Syntaxin-4 fluorescence intensity by the total Myc-Syntaxin-4 fluorescence intensity. **, *p* < 0.0025. *C*, representative images of surface expression level of SEP-GluK2 in active form Rab17 and *Stx4*-shRNA-transfected neurons. At 8 DIV, rat hippocampal neurons were transfected with pCDNA3-SEP-Myc-GluK2 together with pCAG-Control and pFIV-Control, pCAG-Control, and pFIV-shStx4-1, pCAG-Myc-Rab17-Q77L, and pFIV-Control, or pCAG-Myc-Rab17-Q77L and pFIV-shStx4-1. At 11 DIV, the neurons were fixed and subjected to immunocytochemistry with antibodies against GFP (surface, *red* and total, *green*) and Rab17 (*black*). *Bar,* 10 μm. *D,* quantification of the surface expression level of SEP-GluK2 of control neurons (*n* = 10), *Stx4*-shRNA-1-transfected neurons (*n* = 10), Rab17-Q77L-transfected neurons (*n* = 10), and Rab17-Q77L- and *Stx4*-shRNA-1-transfected neurons (*n* = 10) as shown in *C*. Surface expression level of SEP-GluK2 was calculated by dividing the surface SEP-GluK2 fluorescence intensity by the total SEP-GluK2 fluorescence intensity. *A.U.,* arbitrary units. *, *p* < 0.025; **, *p* < 0.0025.

Rab17 is translocated from the soma to the dendrites in a GTP-dependent manner (26). Furthermore, expression of a constitutively active form of Rab17 (Rab17-Q77L) significantly enhances dendrite growth in early stage neurons (18). We therefore investigated whether constitutively active Rab17 increases dendritic Syntaxin-4. Overexpression of EGFP-Rab17-Q77L enhances dendritic, but not axonal, levels of Syntaxin-4 in 11 DIV hippocampal neurons (Myc-Syntaxin-4 in the dendrites; EGFP, 34.2 ± 3.9% *versus* EGFP-Rab17-Q77L, 59.6 ± 3.7%; Fig. 8, *A* and *B*). We also tested whether Rab17 promotes GluK2 membrane insertion in developing neurons. Constitutively active Rab17 increased surface expression of Super-ecliptic pHluorin (SEP)-tagged GluK2 (Rab17-Q77L, 342.0 ± 36.4% of SEP-GluK2 of the control neurons; Fig. 8, *C* and *D*). Moreover, this enhancement was almost completely blocked by co-transfection with *Syntaxin-4*-shRNA (Rab17-Q77L and *Stx4*-shRNA-1, 122.5 ± 19.2% of Myc-GluK2 of the control neurons; Fig. 8, *C* and *D*). These data indicate that Syntaxin-4 works downstream of Rab17 to the surface insertion of GluK2.

##### Syntaxin-4 Is Not Involved in Dendrite Morphogenesis at Developing Hippocampal Neurons

**FIGURE 9.**
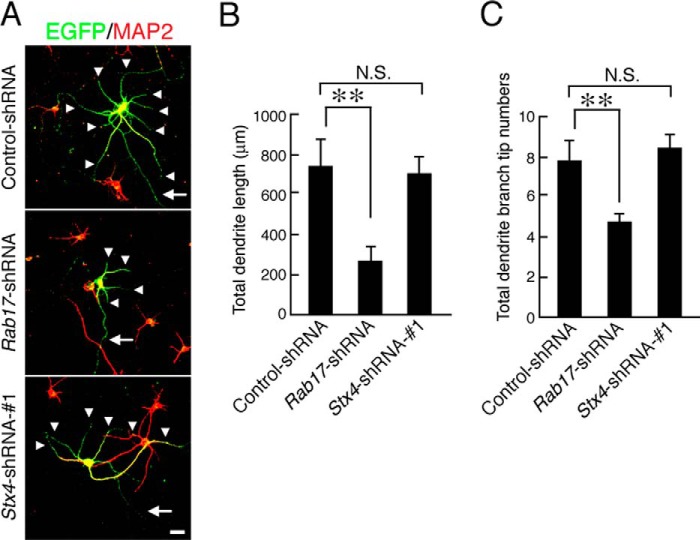
**Syntaxin-4 is not required for the control of dendritic morphogenesis.** *A,* typical images of Rab17- and Syntaxin4-knockdown neurons. At 8 DIV, hippocampal neurons were transfected with a vector encoding EGFP and control-shRNA (*upper panel*), *Rab17*-shRNA (*middle panel*), or *Stx4*-shRNA-1 (*lower panel*), and at 11 DIV, the neurons were fixed and subjected to immunocytochemistry with antibodies against MAP2 (*red*). The *arrows* and *arrowheads* point to axons and dendrites, respectively. *Bar,* 50 μm. *B* and *C,* quantification of the total dendrite length (*B*) and total dendrite branching tip numbers (*C*) of the control neurons (*n* = 20), Rab17-knockdown neurons (*n* = 20), and Syntaxin-4-knockdown neurons (*n* = 20). **, *p* < 0.0025. *N.S.*, not significant.

Because Rab17 regulates dendritic morphogenesis in developing mouse hippocampal neurons (18), we examined whether Syntaxin-4 is also involved in this process. Knockdown of Rab17 reduced the total dendrite length and number of dendrite branches (to 36.7 ± 10.1 and 58.6 ± 10.2%, respectively, of the control; Fig. 9, *A–C*) in 11 DIV hippocampal neurons, but knockdown of Syntaxin-4 did not affect both total dendrite length and number of dendrite branches. Thus, unlike Rab17, Syntaxin-4 does not appear to play a role in dendrite morphogenesis in developing hippocampal neurons.

## DISCUSSION

**FIGURE 10.**
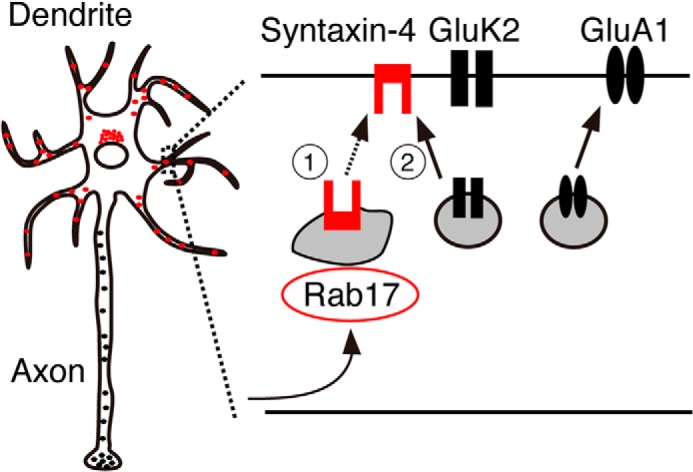
**Schematic representation of proposed roles of Rab17.** Rab17 mediates polarized trafficking of Syntaxin-4, which controls GluK2 but not GluA1 surface insertion at the dendrite in hippocampal neurons.

Our results identify Rab17 as an important regulator of GluK2-containing KAR but not GluA1-containing AMPAR surface expression in cultured rat hippocampal neurons. We show that Rab17 is involved in the polarized localization of Syntaxin-4, which in turn regulates GluK2 trafficking. As represented schematically in Fig. 10, we interpret these data to suggest that Syntaxin-4 is a cargo molecule of Rab17-containing vesicles and that it is an important determinant of KAR insertion in dendritic membrane.

Several Rab proteins have been reported to directly transport AMPAR- or KAR-containing vesicles (10, 12). However, because Rab17 is the only reported dendrite-specific Rab and is involved in dendritic morphogenesis and postsynaptic development (18), we tested whether it is also involved in receptor forward trafficking. We showed that the AMPAR subunits GluA1 and GluA2 do not co-localize with Rab17 (18). Similarly, here we show that the KAR subunit GluK2 also does not co-localize with Rab17 (Fig. 2*G*), indicating that AMPAR- or KAR-containing vesicles do not contain Rab17. Nonetheless, we show that Rab17 and Syntaxin-4 contribute to GluK2 (but not GluA1) surface insertion at the dendrite in developing hippocampal neurons. Given the dynamic regulation of KAR surface expression (13, 23, 27), we expect that future studies will investigate the activity dependence of Rab17 and Syntaxin-4 involvement in processes.

Because GluK2 is predominantly surface-expressed in dendrites in our experiments (Fig. 2, *A* and *B*), we have not determined whether Rab17 and Syntaxin-4 also regulate presynaptic KAR. Extensive future studies will be necessary to determine the transport, insertion, and internalization mechanism of AMPARs and KARs at the dendrite and axon.

Syntaxin-4 is present in dendritic spines in hippocampal neurons (8), but there are significant discrepancies between reports regarding the physiological function of Syntaxin-4 and AMPAR regulation. Kennedy *et al.* (8) used fluorophore-tagged transferrin receptor (TfR-SEP) as a marker for GluA1 trafficking and reported that Syntaxin-4 knockdown or overexpression of a dominant-negative Syntaxin-4 prevents activity-dependent surface insertion of TfR-SEP in spines and blocks LTP. Based on these observations, they concluded that Syntaxin-4 is involved in the synaptic surface insertion of AMPAR. In direct contrast however, Jurado *et al.* (9) state that knockdown of Syntaxin-3, but not of Syntaxin-4, inhibits surface insertion of endogenous GluA1 and blocks LTP (9). In agreement with Jurado *et al.* (9), our data indicate that Syntaxin-4 is not involved in AMPAR trafficking. Nonetheless, because KARs are implicated in postsynaptic LTP (28), Syntaxin-4 may still contribute to this process.

We interpret our results to suggest that Syntaxin-4 is a cargo molecule of Rab17-containing vesicles, which are specifically sorted for polarized trafficking to the dendrite in developing hippocampal neurons. Although we detected mis-trafficked axonal Myc-Syntaxin-4 in Rab17 knockdown neurons (Fig. 5, *D* and *F*), we did not observe similar mislocalization of endogenous Syntaxin-4 (Fig. 6*I*). One explanation for this is that the comparatively low levels of mislocalized endogenous Syntaxin-4 in axons is rapidly degraded, which occur less quickly for the much higher levels of overexpressed Myc-Syntaxin-4. However, future studies will be necessary to determine whether this is correct and define the mechanisms underlying Syntaxin-4 polarization.

Syntaxin-4 is known to be involved in recycling via the endosomal pathway (29, 30), and Rab17 co-localizes with Rab11 in dendritic puncta (18). Furthermore, Syntaxin-4 directly interacts with GTP-bound form of Rab4 (31). Thus, an attractive hypothesis is that Rab17 directly interacts with Syntaxin-4 for sorting into the dendrite-specific recycling endosome at the soma.

Neurons lacking Rab17 have fewer and shorter dendrites and are deficient in dendritic filopodia and spines (18), and kainate stimulation alters dendrite length, branching, filopodia number, and elongation (32–34). However, Syntaxin-4 is not involved in dendrite morphogenesis (Fig. 9). Therefore, we conclude that Syntaxin-4-mediated GluK2 trafficking is not involved in Rab17-dependent dendrite morphogenesis. We speculate that not Syntaxin-4 but instead other cargo molecules of Rab17 or membrane additions from Rab17-containing vesicles to the plasma membrane are important for dendritic morphology in developing hippocampal neurons.

In conclusion, Syntaxin-4 is a cargo molecule of Rab17 in hippocampal dendrites, and both Rab17 and Syntaxin-4 regulate surface insertion of GluK2.

## Footnotes

3 The abbreviations used are:

AMPAR: AMPA-type glutamate receptor
chem-LTP: chemically induced LTP
DIV: days *in vitro*
ECS: extracellular solution
EGFP: enhanced green fluorescent protein
KAR: kainate-type glutamate receptor
LTP: long term potentiation
PFA: paraformaldehyde
SEP: Super-ecliptic pHluorin
shRNA: short hairpin RNA
SR: shRNA-resistant.
